# Effect of Melatonin Plus Zinc Supplementation on Fatigue Perception in Myalgic Encephalomyelitis/Chronic Fatigue Syndrome: A Randomized, Double-Blind, Placebo-Controlled Trial

**DOI:** 10.3390/antiox10071010

**Published:** 2021-06-23

**Authors:** Jesús Castro-Marrero, Maria-Cleofé Zaragozá, Irene López-Vílchez, José Luis Galmés, Begoña Cordobilla, Sara Maurel, Joan Carles Domingo, José Alegre-Martín

**Affiliations:** 1 Division of Rheumatology, ME/CFS Unit, Vall d’Hebron Hospital Research Institute, 08035 Barcelona, Spain; 2 Clinical Research Department, Laboratorios Viñas, 08012 Barcelona, Spain; czaragoza@vinas.es (M.-C.Z.); ilopez@vinas.es (I.L.-V.); jlgalmes@vinas.es (J.L.G.); 3 Department of Biochemistry and Molecular Biomedicine, Faculty of Biology, University of Barcelona, 08028 Barcelona, Spain; bgcordobilla07@ub.edu (B.C.); jcdomingo@ub.edu (J.C.D.); 4 Department of Neurosciences, University of the Basque Country, 48940 Leioa, Spain; saranieves.maurel@ehu.eus; 5 Division of Rheumatology, ME/CFS Unit, Vall d’Hebron University Hospital, 08035 Barcelona, Spain; jalegre@vhebron.net

**Keywords:** chronic fatigue syndrome, fatigue, myalgic encephalomyelitis, melatonin, quality of life, sleep quality, zinc

## Abstract

Myalgic encephalomyelitis/chronic fatigue syndrome (ME/CFS) is a complex, multisystem, and profoundly debilitating condition, probably of multifactorial etiology. No effective approved drugs are currently available for its treatment. Several studies have proposed symptomatic treatment with melatonin and zinc supplementation in chronic illnesses; however, little is known about the synergistic effect of this treatment on fatigue-related symptoms in ME/CFS. The primary endpoint of the study was to assess the effect of oral melatonin plus zinc supplementation on fatigue in ME/CFS. Secondary measures included participants’ sleep disturbances, anxiety/depression and health-related quality of life. A proof-of-concept, 16-week, randomized, placebo-controlled, double-blind trial was conducted in 50 ME/CFS patients assigned to receive either oral melatonin (1 mg) plus zinc (10 mg) supplementation (*n* = 24) or matching placebo (*n* = 26) once daily. Endpoint outcomes were evaluated at baseline, and then reassessed at 8 and 16 weeks of treatment and 4 weeks after treatment cessation, using self-reported outcome measures. The most relevant results were the significant reduction in the perception of physical fatigue in the Mel-Zinc group at the final treatment follow-up versus placebo (*p* < 0.05), and the significant improvement in the physical component summary at all follow-up visits in the experimental group. Urinary 6-sulfatoxymelatonin levels were significantly elevated though the treatment in experimental group vs. placebo (*p* < 0.0001); however, no significantly differences were observed for zinc concentration among participants. Our findings suggest that oral melatonin plus zinc supplementation for 16 weeks is safe and potentially effective in reducing fatigue and improving the quality of life in ME/CFS. This clinical study was registered on ClinicalTrials.gov (NCT03000777).

## 1. Introduction

Myalgic encephalomyelitis, also termed chronic fatigue syndrome (ME/CFS), is a complex debilitating condition with no known etiology. Its hallmark symptoms are severe and disabling physical and mental fatigue linked to post-exertional malaise, which does not improve with rest and seriously interferes with work activity and daily life tasks. To date, no diagnostic tests or biomarkers have been established for ME/CFS, nor does any universally effective treatment exist [1].

Recently, our group demonstrated a significant association between sleep quality, assessed through the Pittsburgh Sleep Quality Index (PSQI) questionnaire, and other clinical symptoms (fatigue, pain and psychopathology) using self-reported measures in patients with ME/CFS [2]. Beyond the diagnosis of ME/CFS, it is important to assess fatigue perception through the Fatigue Impact Scale (FIS-40) [3], quality of life through a health-related quality of life questionnaire (SF-36) [4] and anxiety and depression symptoms using the Hospital Anxiety and Depression Scale (HADS) [5]. The non-pharmacological treatment approaches of ME/CFS, based on scientific evidence, includes cognitive behavioral therapy and graded exercise therapy, and pharmacological treatment is administered to address the core symptoms such as pain, memory/concentration problems, anxiety and depression, orthostatic intolerance and sleep disturbances [6].

Alterations in redox metabolism homeostasis and bioenergetic status, and mitochondrial, neuroimmune and neurovegetative dysfunctions have been implicated in the etiopathogenic hypotheses of ME/CFS [7]. A detailed review of the literature suggests several marginal nutritional deficiencies, which may be of etiological relevance. These include deficiencies of several B-complex vitamins (primarily riboflavin, thiamine, and pyridoxine), vitamin C, magnesium, sodium, melatonin, zinc, L-tryptophan, L-carnitine, Coenzyme Q10, and essential fatty acids (omega-3 PUFAs such as EPA and DHA) [8,9,10].

Melatonin is an endogenous hormone whose concentrations vary according to the day/night circadian cycle. It is produced primarily in the pineal gland, predominantly at night and participates in a wide variety of cellular, neuroendocrine, and neurophysiological processes through membrane receptors (MT1, MT2) and nuclear receptors. In addition to its hypnoinductive action, melatonin is a powerful antioxidant and reactive oxygen and reactive nitrogen species scavenger that reduces oxidative stress and inhibits the formation of free radicals. It also stimulates the immune system; it has receptors expressed on helper T lymphocytes (CD4+ and CD8+ T cells) that produce IL-4, which, in turn, triggers IgA production in B cells. It stimulates cytotoxic phagocytes and T lymphocytes and stabilizes circadian rhythms [11]. In this regard, our work group has reported severe alterations in the circadian rhythm and autonomic dysfunction from individuals with ME/CFS [12]. Therefore, support for the possible use of melatonin in ME/CFS is growing.

Zinc is an essential trace element, which is present in more than 300 specific metalloenzymes, playing an essential role in their activation or structural stability, in addition to serving as a structural ion in more than 200 transcription factors. Zinc plays a critical role in synaptic neuroplasticity and learning [13]. It is needed to achieve a balanced immune function, reducing pro-inflammatory cytokines and decreasing oxidative stress [14], and its use has been also shown to be effective in various depressive processes [15].

To date, symptomatic treatment using oral melatonin plus zinc supplementation in individuals with ME/CFS has not been evaluated. Effective treatment alternatives for ME/CFS are lacking, and the treatments available at present are mostly limited to providing partial symptom relief [6].

Nutraceutical supplementation using melatonin is receiving increasing attention as a potential therapeutic intervention in sleep disturbances of fatiguing chronic diseases. It has shown a good safety profile and promising clinical effects, but to date, it has not been extensively assessed in ME/CFS [16,17]. Here, we describe a first pilot trial to evaluate the clinical response after oral melatonin plus zinc supplementation on the perception of fatigue, sleep disturbances, anxiety and depression and health-related quality of life in individuals with ME/CFS.

## 2. Materials and Methods

### 2.1. Study Participants

**Figure 1:**
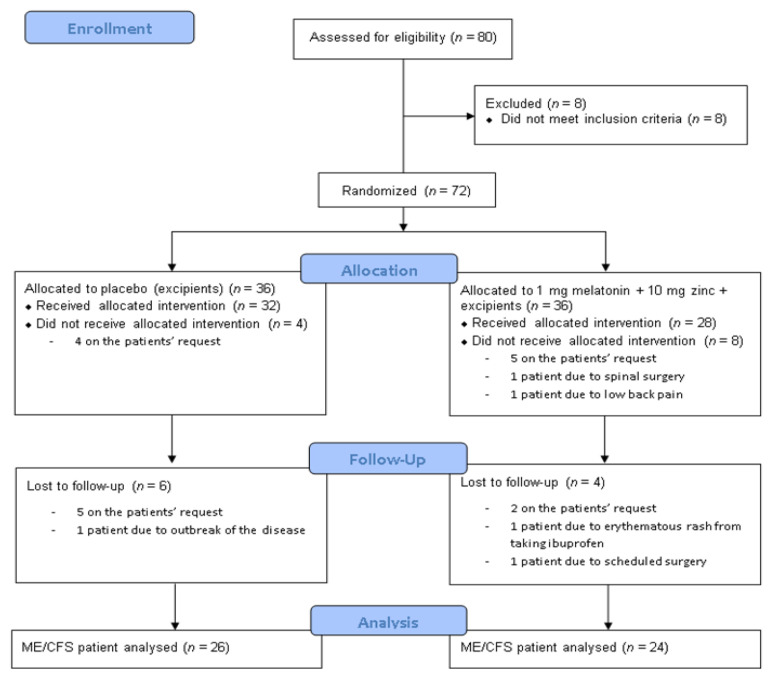
Consolidated Standards of Reporting Trials (CONSORT) flow diagram.

A 16-week randomized, double-blinded, placebo-controlled pilot study was conducted in 80 Caucasian ME/CFS patients consecutively recruited from a single outpatient tertiary referral center (ME/CFS Unit, Vall d’Hebron University Hospital, Barcelona, Spain) from May 2016 (first patient inclusion) to August 2017 (last clinical visit). Figure 1 summarizes the flowchart of the participants prior to analysis. Patients were eligible for the study if they were female, aged between 18 and 65 years, had a confirmed diagnosis of ME/CFS according to the 1994 CDC/Fukuda case definition [18] and provided signed informed consent. Exclusion criteria were: any active medical condition that explained chronic fatigue (untreated hypothyroidism, sleep apnea, narcolepsy, medication side-effects), previous diagnosis not unequivocally resolved (chronic hepatitis, malignancy), past or current psychiatric disorders (major depressive disorder with psychotic or melancholic features, bipolar disorder, schizophrenia, delusional disorder, dementias, anorexia nervosa, bulimia nervosa), participation in another clinical trial of the same or different nature in the 30 days prior to study inclusion, in the judgment of the investigator, inability to follow the instructions or to complete the treatment satisfactorily, failure to provide signed informed consent, current consumption of medications that may interfere with the results and/or whose withdrawal may be a relevant problem, anticoagulant treatment, pregnancy or breast-feeding, had not used oral contraceptives or other hormonal preparations in the previous 6 months, smoking, alcohol intake or substance abuse, severe obesity (class 3 BMI ≥ 40 kg/m^2^) and hypersensitivity to melatonin and/or zinc. Patients with missing data from the follow-up visits to baseline were defined as having dropped out.

### 2.2. Intervention

In total, 80 eligible ME/CFS patients were screened, of whom eight were excluded from the study because they did not meet the inclusion criteria. The remaining 72 participants were randomized by an independent investigator not otherwise involved in the intervention, using the table of random numbers in the Milton statistical guide [19]; they were allocated in a double-blind fashion with a 1:1 ratio either to the experimental group (Mel-Zinc, *n* = 36) or matching placebo (*n* = 36).

Twelve subjects did not attend the baseline visit: nine (five in the experimental group and four in the placebo group) of their own accord and three (all from the experimental group) were considered lost due to limited mobility (spinal surgery, ankle sprain and low back pain, respectively). A total of 60 participants (placebo, *n* = 32 and experimental group, *n* = 28) were visited at baseline, but only 50 (83%) completed the final assessment at 20 weeks; three participants (5%) were lost to follow-up (one in the placebo group with flares of the disease, one in the experimental group with erythematous rash from taking ibuprofen and another scheduled for hip surgery) and seven (12%) abandoned the study at their own request (five in the placebo group and two in the experimental group). The remaining ME/CFS patients completed all the study protocol procedures and were included in the analyses of outcome measures (Figure 1).

### 2.3. Tested Product

The fast-release formulation tested contained a homogenized mixture of 1 mg of melatonin, which was manufactured through an organic synthesis process using as primary starting product 5-methoxytryptamine by Flamma group, Italy, and 10 mg of zinc (zinc sulphate 1-hydrate of synthetic origin by Quality Chemicals, Barcelona, Spain), including isomaltose, and magnesium stearate as excipient based on local E.U. regulations (see information at https://www.efsa.europa.eu/es/efsajournal/pub/2241, accessed on 21 April 2021). The matching placebo capsules were made of the excipient only. The mixture was encapsulated in a purple gelatin capsule. Each bottle contained enough capsules for four months of consumption (120 capsules/bottle); posology consisted of one capsule once a day at night (30 min before bedtime) for 16 weeks. The fast-release preparations (experimental and placebo capsules) were identical in size, shape, color, opacity and taste, and also in terms of presentation and packaging to avoid differentiation by the participants or the research staff. The study pharmacist recorded all treatments supplied on the medication-dispensing forms along with the original script. Both treatments were provided by Laboratorios Viñas, S.A. (www.vinas.es, Barcelona, Spain, accessed on 21 April 2021).

### 2.4. Study Design

**Figure 2:**
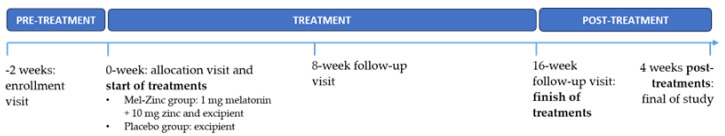
Summarized study schedule at each visit throughout the clinical trial.

This 16-week randomized, double-blinded, placebo-controlled pilot trial was performed at a single outpatient tertiary referral center (ME/CFS Unit, Vall d’Hebron University Hospital, Barcelona, Spain) from May 2016 through August 2017. All participants were Caucasian, had a sedentary lifestyle and were from the same geographical area at the time of study. Clinical visits throughout the study are detailed in Figure 2, which also describes the trial design in both groups. After a verbal description of the study, all participants gave written consent prior to its commencement. Patients were evaluated at baseline and at each follow-up visit (after 8 and 16 weeks of treatment and 4 weeks post-treatment). Changes in symptoms were assessed through self-reported questionnaires completed by participants under the supervision of two trained investigators (J.C.-M. and J.A.) who oversaw compliance. The current clinical study was registered at https://clinicaltrials.gov/ (reference number: NCT03000777, accessed on 21 April 2021).

### 2.5. Primary Outcome Measure

The primary outcome measure was the evaluation of the relief of self-reported fatigue using the 40-item Fatigue Impact Scale (FIS-40) questionnaire after oral melatonin plus zinc supplementation in individuals with ME/CFS. The FIS-40 includes three subscales of the perceived impact of fatigue: cognitive (10 items), physical (10 items) and psychosocial functions (20 items), each item being scored from 0 (no fatigue) to 4 (severe fatigue). The total score is calculated by adding together the responses from the 40 questions (range 0–160). Higher scores indicate more functional limitations due to fatigue [3].

### 2.6. Secondary Outcome Measures

The secondary outcome measures included changes in health-related quality of life (HRQoL), sleep disturbances, anxiety and depression through validated self-reported questionnaires.

#### 2.6.1. The Short-Form 36-Item Health Survey

The 36-item short-form health survey (SF-36) was used to assess health-related quality of life (HRQoL). The SF-36 is a broadly-based self-reported survey on health-related physical and mental functioning status. It assesses functioning on eight subscales, including domains of physical functioning, physical role, bodily pain, general health, social functioning, vitality, emotional role and mental health, and two general subscales covering the physical and mental health domains on a 0–100 scale. Lower scores indicate a more negative impact of an individual’s health on functioning [4].

#### 2.6.2. Pittsburgh Sleep Quality Index

Sleep disturbances were assessed through the self-administered 19-item Pittsburgh Sleep Quality Index (PSQI) questionnaire. Scores are obtained on each of seven components of sleep quality: subjective sleep quality, sleep latency, sleep duration, habitual sleep efficiency, sleep perturbations, use of sleeping medication and daytime dysfunction. Each component is scored from 0 to 3 (0 = no sleep problems and 3 = severe sleep problems). The global PSQI score ranges from 0 to 21 points, with scores of ≥5 indicating poorer sleep quality [20].

#### 2.6.3. Anxiety and Depression

Severity of symptoms of anxiety and depression were scored using the Hospital Anxiety and Depression Scale (HADS), a validated self-reported tool composed of 14 items (seven related to anxiety symptoms and seven to depression) among participants. Each item on the HADS questionnaire is scored from 0–3, and thus, scores range from 0 to 21; scores of 0–7 are interpreted as normal, 8–10 as mild, 11–14 as moderate and 15–21 as severe for either anxiety or depression. The total HADS score ranges from 0 (no anxiety or depression) to 42 (severe anxiety and depression) [5].

### 2.7. Measurement of Melatonin and Zinc

#### 2.7.1. Specimen Collection and Processing

Participants were instructed to collect their 24-h urine samples directly into the dark-colored sterile container, including HCl as preservative (Ref# 77578, Sarstedt, Barcelona, Spain) the day before each visit. On the day after overnight collection, urine samples were refrigerated before transport to the laboratory. The urine samples were stabilized and the pH was not altered. They then were kept frozen in 10 mL V-monovette tubes (Ref# 112452, Sarstedt, Barcelona, Spain) at −80 °C until further analysis. One aliquot of each participants’ urine sample was thawed and then centrifuged (Eppendorf, Centrifuge 5810, Eppendorf Ibérica, Spain) at 2500 rpm for 15 min at room temperature.

Additionally, after overnight fasting for 12 h, blood samples were also collected from an antecubital vein using a vacutainer system (SST tubes, Becton Dickenson, Sarstedt, Barcelona, Spain) from each participant. Blood samples were allowed to clot at 25 °C for 15 min and then centrifuged (Thermo Fisher Scientific, Heraus Megafuge 40, Langenselbold, Germany) at 1800× *g* for 10 min at 4 °C to obtain serum. On baseline and at 8, 16 and 4 weeks after treatment, 24-h urine and blood samples were taken to measure the concentration of melatonin (as 6-sulphatoxymelatonin aMT6s) and zinc, respectively.

#### 2.7.2. Measurements of 6-Sulphatoxymelatonin and Zinc

The 6-sulphatoxymelatonin (aMT6s) concentration—the major metabolite of melatonin in urine—was measured in duplicated 24-h urine samples from each participant using a commercially available competitive enzyme-linked immunosorbent assay (Ref # RE54031, IBL International GmbH, Hamburg, Germany) according to the manufacturer’s instructions. The assay sensitivity was 1 ng/mL, and the inter-assay and intra-assay coefficients of variation (CV) were 5.1–14.9% and 5.2–12.2%, respectively. The urinary aMT6s concentration was normalized based on the excreted 24-h urine volume as indicated by the participants. To adjust for variation in the urinary dilution, the amount of urinary aMT6s was expressed as ng aMT6s/mL urine.

Zinc content was assayed in each duplicated serum sample (100 μL) using an ESI SC-0400 One Fast system by inductively coupled plasma mass spectrometry (Thermo Scientific XSERIES 2 ICP-MS) at the Echevarne core laboratory (Barcelona, Spain) following the manufactures’ instructions. Each sample was analyzed together with five levels of calibrators, blanks and quality controls, all prepared with an amount of serum equivalent to that of the samples. The intra- and inter-assay coefficients of the variations were below 6%, and the low limit of detection was 80 μg/dL.

### 2.8. Sample Size Estimation

Accepting an alpha risk of 5% and a beta risk of 20% in a two-sided paired test, 40 subjects were necessary in each group to obtain statistically significant differences, expected to be of 5% in the placebo group and 30% in the experimental group. A drop-out rate of 20% was anticipated (https://www.imim.es/ofertadeserveis/software-public/granmo/, accessed on 21 April 2021) [21].

### 2.9. Statistical Analysis

The descriptive analysis was performed on the number of valid cases by means of absolute and relative frequencies, measures of central tendency (mean and median) and dispersion (standard deviation, SD and standard error of the mean, SEM). Data are expressed as the mean ± SEM as appropriate. Baseline demographic and clinical characteristic data were expressed as the mean ± SD and compared by Student’s t-test for continuous variables and by Fisher’s exact test for categorical variables. Normality of the samples was verified by the Shapiro–Wilk test, and intragroup comparisons of continuous variables that followed a normal distribution were performed using Student’s t-test for paired samples. If the hypothesis of normality could not be assumed, the analysis was tested using Wilcoxon’s signed rank test. For each intervention group, the treatment effect was assessed as the mean change in variables from baseline to 8 and 16 weeks (score at 8- or 16-week visit minus score at baseline visit), and the treatment withdrawal effect was assessed as the mean change in variables from 16 weeks to 4 weeks’ post-treatment (score at 4 weeks post-treatment minus the score at 16 weeks post-treatment), and the significance threshold was set at * *p* < 0.05, ** *p* < 0.01 and *** *p* < 0.001. Between-treatment analysis was performed by Wilcoxon’s test at each visit. Data were analyzed in an irreversibly anonymized fashion. Statistical analysis was carried out using the R-Studio desktop statistical software (version 1.3.1093). For all analyses, a *p*-value < 0.05 was considered statistically significant.

## 3. Results

### 3.1. Demographic and Clinical Characteristics of the Study Population

Baseline demographic and clinical characteristics of the study participants are presented in Table 1. No significant differences were observed in age, demographic and clinical data between the two groups at baseline. The placebo group consisted of 26 patients and the melatonin plus zinc group of 24 patients.

### 3.2. Primary Outcome Measure

**Figure 3:**
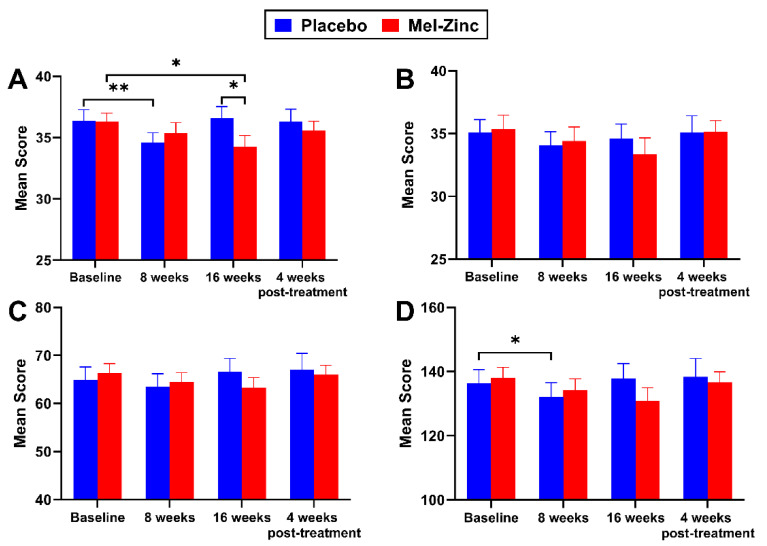
Changes in the FIS-40 domain scores during the intervention study. Each bar indicates the mean FIS-40 item scores ± SEM compared by a paired Student’s t-test, where appropriate, for intragroup analysis and by a Wilcoxon’s signed rank test for intergroup analysis. (**A**) Physical functioning domain; (**B**) Cognitive domain; (**C**) Psychological domain; (**D**) Total FIS-40 score. The significance threshold was set at * *p* < 0.05 and ** *p* < 0.01. No significant differences in any FIS-40 domain scores were observed between the intervention groups in the rest of study visits. Lower scores indicate an improvement in the fatigue perception among participants. Abbreviations: Mel-Zinc, Melatonin plus Zinc group; FIS-40, 40-item fatigue index scale.

Figure 3 shows the fatigue perception scores over the course of the clinical study in the two intervention groups. The perception of physical fatigue (assessed by the physical functioning domain) significantly improved at the 16-week visit in the melatonin plus zinc group (*p* = 0.026) compared to the placebo group. Moreover, in the experimental group, an improvement was observed from the beginning, reaching statistical significance by the end of the treatment (16 weeks vs. baseline, *p* = 0.012), while in the placebo group, the beneficial effect was reached at 8 weeks vs. baseline (*p* = 0.004) but was lost at the final treatment (Figure 3A).

As Figure 3B shows, with regard to the cognitive domain, both groups evolved in parallel; however, unlike the placebo group, the melatonin plus zinc group showed a trend towards improvement that was maintained over the 16 weeks of treatment, although it did not reach statistical significance (16 weeks vs. baseline, *p* = 0.058). The psychological domain evolved in the same way in both groups and over the course of treatment (Figure 3C). Finally, in the placebo group, the FIS-40 total score showed an improvement (*p* = 0.043) at the 8-week visit with respect to baseline; this benefit disappeared at the 16-week visit, returning to baseline scores. In the melatonin plus zinc group, there was a marked improvement at the 16-week visit, though this did not reach significance (16 weeks vs. baseline, *p* = 0.078). The evolution of this parameter during the study did not differ between the groups (Figure 3D). Thus, a gradual improvement in all four domains was observed in the melatonin plus zinc group, which disappeared when treatment was withdrawn. In the placebo group, all four domains worsened at the 16-week visit, while patients were still receiving treatment.

### 3.3. Secondary Outcome Measures

#### 3.3.1. The Short-Form 36-Item Health Survey

Table 2 shows the participants’ scores for the SF-36 questionnaire. In the melatonin plus zinc group, the physical functioning domain worsened significantly after treatment withdrawal (4 weeks post-treatment vs. 16 weeks, *p* = 0.011).

The domains physical role functioning, bodily pain, general health perception, vitality, social role functioning and emotional role functioning did not show relevant differences between the two groups. The mental health status domain evolved in a similar way without relevant changes. Only at the 4 weeks post-treatment visit was a significant worsening observed when melatonin plus zinc supplementation was discontinued (4 weeks post-treatment vs. 16 weeks, *p* = 0.003).

The physical component summary showed better scores in the placebo group at the 8-week visit, but this benefit was lost by the time of the 16-week visit. In the melatonin plus zinc group, statistically significant improvements were observed at both visits during treatment (8- and 16-week visits, *p* = 0.02 and *p* = 0.032 respectively). Between-group analysis did not show differences between the groups. In the mental component summary, no significant differences were observed either within or between the groups.

#### 3.3.2. Pittsburgh Sleep Quality Index

Table 3 displays the participants’ sleep quality scores assessed using the PSQI. As in the primary endpoint, the secondary endpoint PSQI scores in patients supplemented with the melatonin plus zinc showed a progressive decrease that was maintained over the 16 weeks of treatment; in general, the placebo group showed a more irregular evolution, with a certain trend towards higher scores at 16 weeks compared to 8 weeks.

Sleep quality and sleep latency improved in both groups at the 16-week visit compared to baseline (*p* = 0.005 in both components for the experimental group, and *p* = 0.049 and *p* = 0.002, respectively, for the placebo group). In addition, in the melatonin plus zinc group, an effect of treatment discontinuation for 30 days was observed, as sleep latency worsened (*p* = 0.041) compared to the 16-week visit.

In the melatonin plus zinc group, sleep duration, sleep disturbances and sleep medication use worsened after 30 days of treatment withdrawal (4 weeks post-treatment vs. 16 weeks, *p* = 0.008, *p* = 0.006 and *p* = 0.047 respectively). On the other hand, in the placebo group, sleep duration, habitual sleep efficiency, sleep disturbances and daytime dysfunction improved at the 8-week visit with respect to baseline (*p* = 0.015, *p* = 0.0001, *p* = 0.008 and *p* = 0.036, respectively), but this benefit was lost at the 16-week visit, returning to baseline levels.

The global PSQI index showed significant improvements in both groups at the 8-week visit (*p* = 0.001 for the placebo group and *p* = 0.018 for the experimental group) and at 16-week visit (*p* = 0.006 for the placebo group and *p* = 0.004 for the experimental group). It also worsened after 4 weeks without treatment in both groups (*p* = 0.037 for the placebo group and *p* = 0.008 for the melatonin plus zinc group).

The between-group analysis did not show differences on any component of the self-administered scale.

Bedtime during the study was between 23:45 ± 01:32 h (earliest time average) and 24:00 ± 01:24 h (latest average time) in the placebo group, and 23:13 ± 01:29 h (earliest time average) and 23:33 ± 01:13 h (latest average time) in the melatonin plus zinc group.

#### 3.3.3. Anxiety and Depression

Table 4 presents the participants’ anxiety and depression scores assessed through HADS. Anxiety improved at the 8-week visit in the placebo group (*p* = 0.004), although the scores had returned to baseline levels by the time of the 16-week visit. Paradoxically, there was an improvement after treatment withdrawal (4 weeks post-treatment vs. 16 weeks, *p* = 0.041). There were no changes in the melatonin plus zinc group.

Depression showed no significant changes between visits in either group. The total HADS score improved at the 8-week visit in the placebo group (*p* = 0.028), although it rose again to baseline values at the 16-week visit. In the melatonin plus zinc group, there were no changes. The between-group analysis did not show differences in any of the domains of the self-administered scale.

#### 3.3.4. Levels of 6-Sulphatoxymelatonin and Zinc among the Participants

Data on the levels of urinary 6-sulphatoxymelatonin (aMT6s) and serum zinc are presented in Table 5. Urinary aMT6s levels were statistically significant at 8 and 16 weeks of treatment in the experimental group compared to placebo (both *p* < 0.0001), thus demonstrating an increase in circulating melatonin levels due to treatment. No statistical differences were found 1 month post-treatment between both groups (*p* = 0.078). We further measured the participants’ serum zinc levels after melatonin plus zinc co-supplementation. No differences were observed in the evolution of zinc levels either over the course of the visits or between the treatment groups (Table 5).

#### 3.3.5. Safety and Tolerability

We report the collective safety data for oral melatonin plus zinc supplementation study in patients with ME/CFS. Few adverse effects have been related to melatonin [22] and zinc [23]. In our study, no relevant treatment-related adverse events were recorded among study participants. These data demonstrate that the oral administration of melatonin plus zinc has a manageable safety and tolerability profile in people with ME/CFS.

## 4. Discussion

Research on the use of nutraceutical interventions as antioxidant supplements to reduce increased oxidative stress in individuals with ME/CFS remains controversial. Current evidence suggests that melatonin and zinc administration may achieve improvements in fatigue perception and health-related quality of life in these patients. In the present study, we failed to detect relevant improvements in sleep quality and anxiety and depression. These results were unexpected, given that melatonin is the regulator of the circadian rhythm, and zinc has been shown to be useful in the treatment of anxiety/depression symptoms [15,24].

Several biochemical and immune abnormalities in inflammatory, oxidative and nitrosative stress pathways have been documented in ME/CFS, and recent studies have suggested that mitochondrial disturbances to energy requirements may be associated with its pathogenesis [7]. There is currently no treatment that modifies the natural evolution to chronicity, and thus, research efforts should focus on finding new molecules that improve the quality of life of these patients. As several nutritional deficiencies have been demonstrated in patients with ME/CFS, many attempts have been made to find therapeutic targets within natural nutritional supplements, although the results have been inconsistent [8,16].

The lack of a straightforward definition and clear-cut criteria for ME/CFS makes it difficult to compare the results of previous studies. The significant overlapping with other fatiguing diseases has caused major biases in many clinical trials for ME/CFS. Furthermore, since nutritional interventions have only recently been acknowledged as a valid part of the therapeutic approach, the body of evidence on their use is limited. Most of the trials conducted to date have been observational, and the interventional trials performed are not always of high quality; few well-conducted randomized controlled trials (RCT) have been performed in patients with ME/CFS. Another problem lies in the nature of nutritional supplementation, which may involve a single nutrient, or the combination of two nutrients with a synergistic biological function, or even cocktails of nutrients, making it even more difficult to analyze and draw conclusions [11]. Alterations in the levels of melatonin and zinc have been described in individuals with ME/CFS and fibromyalgia [25,26,27,28]. To our knowledge, this is the first randomized, placebo-controlled, double-blind trial to evaluate the potentially beneficial effects of oral melatonin plus zinc supplementation due to their potential synergistic antioxidant and anti-inflammatory effects. A previous study conducted by our group in people with ME/CFS evaluated the combination of CoQ10 and nicotinamide adenine dinucleotide (NADH), with a synergistic antioxidant effect, and showed an improvement in the maximum heart rate and in the self-reported fatigue perception after an exercise challenge test (2-day repeated cardiopulmonary exercise test) [29].

Our results suggest that melatonin and zinc supplementation may have a positive effect on the perception of fatigue, as both intragroup (*p =* 0.012) and intergroup analyses (*p =* 0.026) showed statistically significant improvements in the FIS-40 physical domain after 16 weeks of treatment (Figure 3A). Likewise, our nutrient combination may have influenced the FIS-40 cognitive domain, with a progressive improvement at the end of the treatment, which almost reached statistical significance (Figure 3B, *p* = 0.058), and also the overall FIS-40 score (Figure 3D, *p* = 0.079). In view of the positive trend found for the melatonin plus zinc group, we consider that this study should be continued with a larger number of ME/CFS patients, taking gender into consideration. In the experimental group, the physical functioning item worsened significantly after treatment withdrawal compared to the 16-week follow-up visit among participants (Table 3, *p* = 0.011), indicating a direct benefit associated with the melatonin plus zinc treatment.

In a study of melatonin in ME/CFS patients with delayed circadian rhythmicity, van Heukelom et al. [30] found significant improvements after treatment in fatigue, memory/concentration, motivation and functional activity. Pardini et al. [31] conducted an efficacy study between melatonin and agomelatine (melatonin receptor agonist MT1 and MT2) in ME/CFS patients, reporting an improvement in the perception of fatigue in the agomelatine group. Starreveld et al. [32] found that light therapy improves chronic fatigue in (non-) Hodgkin lymphoma survivors through action on the suprachiasmatic nucleus, which stimulates the release of melatonin. Steur et al. [33] showed that the fatigue associated with acute lymphoblastic leukemia is related to changes in the sleep–wake rhythm. In addition to these studies, our group has recently demonstrated that ME/CFS patients present significant alterations in circadian rhythm patterns [12]. In our study, besides the benefit in physical fatigue, we observed a worsening in patient-reported PSQI domains four weeks after withdrawal of treatment with melatonin plus zinc co-supplementation in the study participants. Further studies with larger ME/CFS cohorts, including longer treatment periods with melatonin and zinc and measurement of biomarkers of circadian rhythm (nocturnal 24-h urine and plasma melatonin levels) and dysautonomia before and after an exercise challenge test (2-day consecutive cardiopulmonary exercise test) in ME/CFS are now required.

Previous reports have suggested that dietary supplements, such as CoQ10 and NADH, are safe and well tolerated among ME/CFS patients, just as we found in our study with the combination of melatonin plus zinc. The most frequently reported adverse effects were daytime sleepiness, headache, and dizziness [22]. In fibromyalgia, an entity included within the central sensitization syndromes and ME/CFS-associated comorbid condition, several studies have been carried out with melatonin administration and have reported reductions in pain and improvements in sleep problems and healthy-related quality of life [34]. In multiple sclerosis, an entity with an immunoinflammatory basis in which fatigue plays an important role, the administration of melatonin has also been shown to improve fatigue and quality of life through the reduction of oxidative stress markers [35,36].

With regards to zinc, decreased serum zinc concentrations have been identified as a biochemical marker of post-viral fatigue syndrome, depression and activation of the virus-induced immune and inflammatory response [24]. Early clinical manifestations of lowered zinc status include fatigue, depression and cognitive disorders [15]. Previous research has studied the relationship between zinc and chronic fatigue. Maes et al. [25] found a significant relationship between low zinc levels in serum and the values of the α-2 protein fraction and of T-cell activation markers in ME/CFS patients; this result could be explained by a reduction in the zinc levels caused by the sequestration of the intracellular heavy metal binding protein metallothionein in the liver, which, in turn, may be related to an increase in activity of IL-1β and IL-6; in a study of patients with colorectal cancer supplemented with zinc, de Figueiredo Ribeiro et al. [37] observed that fatigue was prevented and quality of life was maintained during chemotherapy. In our study, serum zinc levels in ME/CFS patients were within normal limits [24], and no differences were observed in the evolution of circulating zinc levels, either over the course of the visits or between the two groups.

Our literature search did not reveal any previous studies of supplementation with melatonin plus zinc in individuals with ME/CFS. However, it is known that the combined therapy of melatonin, vitamin C and zinc in other chronic diseases is more effective than the use of these agents alone, due to the synergistic effect of their action to modulate the oxidative stress and immune and neuroinflammatory response [17]. In patients with primary insomnia treated with melatonin, zinc and magnesium supplementation, Rondanelli et al. [38] reported an improvement in sleep quality and in health-related quality of life; additionally, a pre-clinical study in rats demonstrated that increased lipid peroxidation in muscle tissue due to ischemia-reperfusion injury may be prevented by melatonin and/or zinc supplementation [39].

The current study has several limitations that should be mentioned. Firstly, the sample size was relatively small (*n* = 50), and only women participated. While we applied a within-subjects design, future studies should include larger samples and should explore sex-related differences. However, it should also be noted that recent studies have highlighted the utility of smaller samples with more robust measurement designs. Secondly, the sample was derived from the recruitment site of the ME/CFS cohort. As all patients were recruited from a single tertiary referral center (unicenter study), the proportion of more severely ill patients may have been greater than normal, and thus, we should be cautious about generalizing the results to patients seen in other general healthcare settings (i.e., primary and/or outpatient care) or to the general population. Thirdly, timing and doses were pre-established, and thus, the dose-response effect cannot be analyzed. It might be useful to consider longer interventions (more than 6–12 months) and higher dosages of melatonin and zinc in order to determine their potential beneficial effects. Finally, we did not control for confounding factors, such as comorbidities, diets, habits and lifestyles, among participants. We have no reason to believe that their daily diets and/or lifestyle changed between sessions, but future prospective cohort intervention studies should include dietary habit tracking reports or other accounts to examine these important lifestyle factors in the ME/CFS population.

Our study also has several important strengths. First, it is the first pilot study to assess the effect of oral melatonin plus zinc supplementation in ME/CFS. Second, the combination of melatonin plus zinc improved the perception of physical fatigue and physical quality of life in ME/CFS patients. Third, the withdrawal of the nutritional supplement had a deleterious effect on the sleep quality and health-related quality of life in the study population. Fourth, the use of strict inclusion criteria based on the 1994 CDC/Fukuda case definition for ME/CFS ensured that the participants were appropriately selected and did not have confounding psychiatric comorbidities. Fifth, the combination of melatonin plus zinc was safe and well tolerated among participants. Larger multicenter trials with longer follow-up interventions in more homogeneous ME/CFS populations, examining not only melatonin and zinc levels but also immune and inflammatory response biological markers and the redox system, are now warranted to assess these findings and to produce evidence-based guidelines regarding the potential beneficial effects of antioxidant therapy in ME/CFS and in other fatiguing chronic conditions.

## 5. Conclusions

To the best of our knowledge, this is the first pilot study to assess the effect of melatonin plus zinc supplementation on fatigue perception in ME/CFS patients. We found that oral melatonin plus zinc supplementation significantly improved the perception of physical fatigue and health-related quality of life in ME/CFS patients after 16 weeks of treatment. A treatment withdrawal effect was observed in the melatonin plus zinc group, with symptomatic relapse in the sleep parameters and in the physical function and mental health dimensions (SF-36), but not in the placebo group. Based on these results, the administration of 1 mg of melatonin and 10 mg of zinc daily may be indicated as adjuvant treatment for ME/CFS patients to improve their fatigue and HRQoL. Additional well-powered intervention studies should be conducted to attain a full assessment of the antioxidant and anti-inflammatory effects of oral melatonin and zinc co-supplementation on core symptoms, chronobiologic pathomechanisms and dysautonomia in ME/CFS.

## Figures and Tables

**Table 1:** Baseline demographic and clinical characteristics of study participants who completed the final assessment.

Characteristics	Placebo (*n* = 26)	Mel-Zinc (*n* = 24)	*p*-Value
Age (years)	53.7 ± 9.6	51.0 ± 10.2	0.339
Marital status			0.473
Married	15 (58)	16 (70)	
Single	7 (27)	6 (26)	
Separated/divorced	4 (15)	1 (4)	
BMI (kg/m^2^)	28 ± 0.1	26 ± 0.3	0.651
Systolic BP (mmHg)	118 ± 17.1	114 ± 13.7	0.291
Diastolic BP (mmHg)	78 ± 11.9	72 ± 9.1	0.088
Heart rate (bpm)	75 ± 8	76 ± 12	0.680
Illness duration (years)			0.201
>10	17 (65)	19 (79)	
≤10	6 (23)	4 (17)	
History of chronic pain	25 (96)	24 (100)	0.319
Illness-affected relatives	11 (42)	5 (21)	0.135
Concomitant drugs			
Anticonvulsants	13 (50)	18 (75)	0.217
Antidepressants	19 (73)	24 (85)	0.065
Anxiolytics	5 (19)	6 (25)	0.302
NSAID	12 (46)	11 (46)	0.720
Opioids	9 (34)	12 (50)	0.547

Data are expressed as the mean ± SD for continuous variables and compared by Student’s *t*-test, and categorical variables are given as numbers with percentages (%) and compared by Fisher’s exact test. Abbreviations: Mel-Zinc, Melatonin plus Zinc group; BMI, body mass index; BP, blood pressure; NSAID, non-steroidal anti-inflammatory drugs.

**Table 2:** Health-related quality of life (SF-36 questionnaire) in participants completing final assessment.

SF-36 Domains	Placebo (*n* = 26)	Mel-Zinc (*n* = 24)	*p*-Value ^1^
Physical functioning			
Baseline	23.96 ± 3.84	22.92 ± 3.43	0.893
8 weeks	26.04 ± 4.15	21.04 ± 3.24	0.534
16 weeks	21.75 ± 3.84	26.09 ± 3.46	0.316
4 weeks post-treatment	26.14 ± 4.21	19.77 ± 3.29 *	0.421
Physical role functioning			
Baseline	0.00 ± 0.00	1.04 ± 1.02	0.298
8 weeks	6.00 ± 2.99	1.09 ± 1.04	0.179
16 weeks	3.41 ± 3.20	8.70 ± 5.14	0.323
4 weeks post-treatment	2.08 ± 1.41	4.35 ± 3.25	0.930
Bodily pain			
Baseline	14.35 ± 3.36	13.50 ± 2.75	0.848
8 weeks	20.08 ± 3.72 *	16.79 ± 2.52	0.797
16 weeks	17.35 ± 3.14	17.22 ± 3.41	0.847
4 weeks post-treatment	15.17 ± 3.33	13.78 ± 2.26	0.886
General health perception			
Baseline	14.50 ± 2.64	22.91 ± 2.23	0.015
8 weeks	21.23 ± 4.12 *	24.04 ± 2.98	0.197
16 weeks	15.55 ± 3.33	25.77 ± 3.29	0.017
4 weeks post-treatment	19.18 ± 3.29	22.05 ± 2.89	0.308
Vitality			
Baseline	18.59 ± 4.57	12.99 ± 2.83	0.678
8 weeks	23.40 ± 5.00	15.83 ± 3.81	0.370
16 weeks	16.01 ± 4.19	19.28 ± 4.65	0.482
4 weeks post-treatment	20.00 ± 4.79	18.64 ± 4.47	0.935
Social role functioning			
Baseline	32.69 ± 6.00	28.65 ± 3.57	0.835
8 weeks	41.35 ± 5.33 *	29.69 ± 3.89	0.154
16 weeks	31.52 ± 4.99	34.24 ± 4.14	0.488
4 weeks post-treatment	35.94 ± 6.48	32.07 ± 4.87	0.838
Emotional role functioning			
Baseline	46.15 ± 9.06	42.03 ± 8.81	0.790
8 weeks	41.03 ± 9.67	25.76 ± 7.68 *	0.403
16 weeks	46.97 ± 10.01	36.23 ± 8.74	0.507
4 weeks post-treatment	43.06 ± 9.92	28.99 ± 8.60	0.304
Mental health status			
Baseline	48.00 ± 4.26	47.63 ± 3.27	0.815
8 weeks	50.15 ± 4.93	45.13 ± 3.44	0.553
16 weeks	47.43 ± 4.14	50.43 ± 3.82	0.447
4 weeks post-treatment	47.91 ± 4.63	40.91 ± 3.78 **	0.368
Physical component summary			
Baseline	20.77 ± 1.14	22.53 ± 1.00	0.210
8 weeks	24.48 ± 1.30 **	24.25 ± 1.16 *	0.856
16 weeks	21.29 ± 1.58	25.12 ± 1.20 *	0.070
4 weeks post-treatment	22.85 ± 1.21	24.38 ± 1.21	0.335
Mental component summary			
Baseline	38.76 ± 2.66	35.94 ± 2.10	0.455
8 weeks	38.14 ± 2.85	33.61 ± 2.04	0.329
16 weeks	37.33 ± 2.83	35.95 ± 2.40	0.778
4 weeks post-treatment	38.52 ± 2.95	32.55 ± 2.55	0.201

Data are expressed as the mean ± SEM and compared by a paired Student’s *t*-test, where appropriate, for intragroup analysis, and by a Wilcoxon’s signed rank test for intergroup analysis. The significance threshold was set at * *p* < 0.05 and ** *p* < 0.01. Abbreviations: Mel-Zinc, Melatonin plus Zinc group; SF-36, 36-item short-form health survey. Higher scores indicate better health-related quality of life. ^1^ *p*-values for between-group analysis.

**Table 3:** Sleep quality (assessed with the PSQI questionnaire) in participants completing the final assessment.

PSQI Domains	Placebo (*n* = 26)	Mel-Zinc (*n* = 24)	*p*-Value ^1^
Subjective sleep quality			
Baseline	2.46 ± 0.12	2.50 ± 0.13	0.692
8 weeks	1.92 ± 0.17 **	1.96 ± 0.20	0.791
16 weeks	1.96 ± 0.24 *	1.83 ± 0.20 *	0.540
4 weeks post-treatment	2.21 ± 0.16	1.91 ± 0.21	0.412
Sleep latency			
Baseline	2.46 ± 0.14	2.08 ± 0.17	0.096
8 weeks	1.88 ± 0.19 **	1.75 ± 0.18	0.589
16 weeks	2.00 ± 0.20 **	1.58 ± 0.19 **	0.128
4 weeks post-treatment	2.29 ± 0.18	1.83 ± 0.20 *	0.080
Sleep duration			
Baseline	1.92 ± 0.21	1.63 ± 0.24	0.366
8 weeks	1.54 ± 0.24 *	1.46 ± 0.23	0.817
16 weeks	1.70 ± 0.21	1.25 ± 0.25	0.172
4 weeks post-treatment	1.83 ± 0.23	1.91 ± 0.22 **	0.791
Habitual sleep efficiency			
Baseline	2.31 ± 0.19	1.83 ± 0.24	0.169
8 weeks	1.50 ± 0.25 ***	1.75 ± 0.25	0.466
16 weeks	1.91 ± 0.27	1.58 ± 0.28	0.524
4 weeks post-treatment	2.17 ± 0.21	2.04 ± 0.23	0.768
Sleep disturbances			
Baseline	2.31 ± 0.14	2.04 ± 0.11	0.110
8 weeks	1.92 ± 0.15 **	2.00 ± 0.10	0.642
16 weeks	2.09 ± 0.16	1.88 ± 0.12	0.312
4 weeks post-treatment	2.13 ± 0.17	2.22 ± 0.10 **	0.825
Sleeping medication use			
Baseline	2.00 ± 0.26	1.83 ± 0.28	0.664
8 weeks	2.19 ± 0.26	1.58 ± 0.28	0.118
16 weeks	1.70 ± 0.28	1.38 ± 0.29	0.487
4 weeks post-treatment	2.25 ± 0.24	2.04 ± 0.27 **	0.722
Daytime dysfunction			
Baseline	2.38 ± 0.17	2.58 ± 0.14	0.404
8 weeks	2.00 ± 0.17 **	2.32 ± 0.15	0.158
16 weeks	2.13 ± 0.22	2.21 ± 0.19	0.991
4 weeks post-treatment	2.33 ± 0.15	2.48 ± 0.15	0.476
Global PSQI score			
Baseline	15.85 ± 0.64	14.50 ± 0.63	0.101
8 weeks	12.96 ± 0.90 **	12.83 ± 0.74 *	0.755
16 weeks	13.48 ± 1.07 **	11.71 ± 0.94 **	0.178
4 weeks post-treatment	15.04 ± 0.94 *	14.43 ± 0.84 **	0.448

Data are expressed as the mean ± SEM and compared by a paired Student’s *t*-test, where appropriate, for intragroup analysis and by a Wilcoxon’s signed rank test for intergroup analysis. The significance threshold was set at * *p* < 0.05, ** *p* < 0.01 and *** *p* < 0.001. Abbreviations: Mel-Zinc, Melatonin plus Zinc group; PSQI, Pittsburgh sleep quality index. Lower scores indicate an improvement in the sleep quality perception. ^1^ *p*-values for between-group analysis.

**Table 4:** Anxiety and Depression scores (HADS questionnaire) of the participants who completed the final assessment.

HADS Domains	Placebo (*n* = 26)	Mel-Zinc (*n* = 24)	*p*-Value ^1^
Anxiety			
Baseline	13.12 ± 0.95	11.92 ± 0.77	0.163
8 weeks	11.08 ± 0.90 **	12.46 ± 0.78	0.275
16 weeks	13.00 ± 1.06	11.67 ± 0.80	0.124
4 weeks post-treatment	12.50 ± 1.06 *	12.48 ± 0.73	0.543
Depression			
Baseline	11.62 ± 1.04	11.71 ± 0.67	0.718
8 weeks	10.77 ± 1.01	11.58 ± 0.86	0.907
16 weeks	11.87 ± 1.03	11.08 ± 0.81	0.353
4 weeks post-treatment	11.54 ± 0.96	11.74 ± 0.81	0.535
Total HADS			
Baseline	25.92 ± 1.38	21.92 ± 1.67	0.062
8 weeks	21.85 ± 1.81 *	24.04 ± 1.44	0.613
16 weeks	24.87 ± 1.99	22.75 ± 1.41	0.201
4 weeks post-treatment	24.04 ± 1.94	24.22 ± 1.34	0.400

Data are expressed as the mean ± SEM and compared by a paired Student’s t-test, where appropriate, for intragroup analysis, and by a Wilcoxon’s signed rank test for between-group analysis. The significance threshold was set at * *p* < 0.05 and **p < 0.01. Abbreviations: Mel-Zinc, Melatonin plus Zinc group; HADS, Hospital anxiety and depression scale. Lower scores indicate an improvement in the anxiety/depression symptoms. ^1^ *p*-values for between-group analysis.

**Table 5:** Levels of 6-sulphatoxymelatonin and zinc of the study participants who completed the final assessment.

Melatonin (as aMT6s), ng/mL urine	Placebo (*n* = 26)	Mel-Zinc (*n* = 24)	*p*-Value ^1^
Baseline	16.55 ± 2.39	24.91 ± 3.43	0.063
8 weeks	16.68 ± 2.44	283.50 ± 8.47 ***	<0.0001
16 weeks	14.85 ± 2.14	284.20 ± 8.45 ***	<0.0001
4 weeks after treatment	13.21 ± 2.18	26.45 ± 5.61 ***	0.078
**Zinc, µg/dL**	**Placebo** **(*n* = 26)**	**Mel-Zinc** **(*n* = 24)**	***p*** **-Value ^1^**
Baseline	114.73 ± 3.94	122.04 ± 6.34	0.676
8 weeks	114.42 ± 4.26	120.96 ± 4.92	0.336
16 weeks	126.62 ± 4.74	129.21 ± 6.28	0.641
4 weeks after treatment	121.45 ± 3.18	127.87 ± 5.18	0.735

Data are expressed as the mean ± SEM and compared by a paired Student’s *t*-test, where appropriate, for intragroup analysis, and by a Wilcoxon’s signed rank test for between-group analysis. The significance threshold was set at *** *p* < 0.001. Abbreviations: Mel-Zinc, melatonin plus zinc group. ^1^ *p*-values for between-group analysis.

## Data Availability

The datasets analyzed during the current study are available from the corresponding author on reasonable request.
